# Functional characterization of NK cells in Mexican pediatric patients with acute lymphoblastic leukemia: Report from the Mexican Interinstitutional Group for the Identification of the Causes of Childhood Leukemia

**DOI:** 10.1371/journal.pone.0227314

**Published:** 2020-01-17

**Authors:** Lucero Valenzuela-Vazquez, Juan Carlos Núñez-Enríquez, Jacqueline Sánchez-Herrera, Elva Jiménez-Hernández, Jorge Alfonso Martín-Trejo, Laura Eugenia Espinoza-Hernández, Aurora Medina-Sanson, Luz Victoria Flores-Villegas, José Gabriel Peñaloza-González, José Refugio Torres-Nava, Rosa Martha Espinosa-Elizondo, Raquel Amador-Sánchez, Jessica Denisse Santillán-Juárez, Janet Flores-Lujano, María Luisa Pérez-Saldívar, Luis Ramiro García-López, Alejandro Castañeda-Echevarría, Francisco Rodríguez-Leyva, Haydeé Rosas-Vargas, Minerva Mata-Rocha, David Aldebarán Duarte-Rodríguez, Omar Alejandro Sepúlveda-Robles, Ismael Mancilla-Herrera, Juan Manuel Mejía-Aranguré, Mario Ernesto Cruz-Munoz

**Affiliations:** 1 Facultad de Medicina, Universidad Autónoma del Estado de Morelos, Cuernavaca, Morelos, Mexico; 2 Unidad de Investigación Médica en Epidemiología Clínica, UMAE Hospital de Pediatría, Centro Médico Nacional (CMN) "Siglo XXI", Instituto Mexicano del Seguro Social (IMSS), Mexico City, Mexico; 3 Servicio de Hematología Pediátrica, Hospital General “Gaudencio González Garza”, Centro Médico Nacional (CMN) "La Raza", IMSS, Mexico City, Mexico; 4 Servicio de Hematología Pediátrica, UMAE Hospital de Pediatría, Centro Médico Nacional (CMN) "Siglo XXI", Instituto Mexicano del Seguro Social (IMSS), Mexico City, Mexico; 5 Servicio de Hemato-Oncologia, Hospital Infantil de México Federico Gómez, Secretaria de Salud (SS), Mexico City, Mexico; 6 Servicio de Hematología Pediátrica, Centro Médico Nacional (CMN) “20 de Noviembre”, Instituto de Seguridad Social al Servicio de los Trabajadores del Estado (ISSSTE), Mexico City, Mexico; 7 Servicio de Onco-Pediatria, Hospital Juárez de México, Secretaria de Salud (SS), Mexico City, Mexico; 8 Servicio de Oncología, Hospital Pediátrico de Moctezuma, Secretaría de Salud del D.F., Mexico City, Mexico; 9 Servicio de Hematología Pediátrica, Hospital General de México, Secretaria de Salud (SS), Mexico City, Mexico; 10 Hospital General Regional No. 1 "Carlos McGregor Sánchez Navarro", IMSS, Mexico City, Mexico; 11 Servicio de Hemato-oncología Pediátrica, Hospital Regional No. 1° de Octubre, ISSSTE, Mexico City, Mexico; 12 Servicio de Pediatría, Hospital Pediátrico de Tacubaya, Secretaría de Salud (SS), Mexico City, Mexico; 13 Servicio de Pediatría, HGR No. 25, IMSS, Mexico City, Mexico; 14 Servicio de Cirugía Pediátrica, HGZ No. 30 IMSS, Mexico City, Mexico; 15 Unidad de Investigación Médica en Genética Humana, UMAE Hospital de Pediatría, Centro Médico Nacional (CMN) "Siglo XXI", IMSS, Mexico City, Mexico; 16 Departamento de infectología e inmunología, Instituto Nacional de Perinatología, Mexico City, Mexico; 17 Coordinación de Investigación en Salud, Instituto Mexicano del Seguro Social (IMSS), Mexico City, Mexico; European Institute of Oncology, ITALY

## Abstract

Acute lymphoblastic leukemia (ALL) is the most common cancer in children around the globe. Mexico City has one of the highest incidence rates of childhood leukemia worldwide with 49.5 cases per million children under the age of 15 which is similar to that reported for Hispanic populations living in the United States. In addition, it has been noted a dismal prognosis in Mexican and Hispanic ALL pediatric population. Although ALL, like cancer in general, has its origins in endogenous, exogenous, and genetic factors, several studies have shown that the immune system also plays a deterministic role in cancer development. Among various elements of the immune system, T lymphocytes and NK cells seem to dominate the immune response against leukemia. The aim of the present study was to perform a phenotypic and functional characterization of NK cells in ALL Mexican children at the moment of diagnosis and before treatment initiation. A case-control study was conducted by the Mexican Interinstitutional Group for the Identification of the Causes of Childhood Leukemia (MIGICCL). 41 cases were incident ALL children younger than 17 years old and residents of Mexico City. 14 controls were children without leukemia, matched by age and sex with cases. NK cell function was evaluated by degranulation assays towards K562 cells and SLAM-associated protein (SAP) expression was measured by intracellular staining. All assays were performed using peripheral blood mononuclear cells from controls and patients. The results indicate that NK mediated cytotoxicity, measured by CD107a degranulation assays in response to K562 cells, was reduced in ALL patients compared to controls. Interestingly, an impaired NK cell killing of target cells was not equally distributed among ALL patients. In contrast to patients classified as high-risk, standard-risk patients did not display a significant reduction in NK cell-mediated cytotoxicity. Moreover, patients presenting a leukocyte count ≥ 50,000xmm^3^ displayed a reduction in NK-cell mediated cytotoxicity and a reduction in SAP expression, indicating a positive correlation between a reduced SAP expression and an impaired NK cell-mediated citotoxicity. In the present study it was observed that unlike patients with standard-risk, NK cells from children presenting high-risk ALL, harbor an impaired cytotoxicity towards K562 at diagnosis. In addition, NK cell function was observed to be compromised in patients with a leukocyte count ≥50,000xmm^3^, where also it was noticed a decreased expression of SAP compared to patients with a leukocyte count <50,000xmm^3^. These data indicate NK cell-mediated cytotoxicity is not equally affected in ALL patients, nevertheless a positive correlation between low SAP expression and decreased NK cell-mediated cytotoxicity was observed in ALL patients with a leukocyte count ≥50,000xmm^3^. Finally, an abnormal NK cell-mediated cytotoxicity may represent a prognostic factor for high-risk acute lymphoblastic leukemia.

## Introduction

Acute lymphoblastic leukemia (ALL) is the most common cancer during childhood. Different clinical, immunophenotypic and molecular subtypes have been described for the disease [1]. In contrast to other cancers, ALL has reached cure rates around 90% in developed countries as a result of the improvement in chemotherapy treatments based on a prognostic stratification. Among prognostic features, the age and white blood cell count (WBC) are the most consistently used across chemotherapy protocols [2].

Mexico City has one of the highest incidence rates of childhood leukemia worldwide with 49.5 cases per million children under the age of 15, which is similar to that reported for Hispanic populations living in the United States [3–5]. In addition, the Hispanic population not only has the highest incidence rates in ALL, but also one of the lowest survival rates within the United States, so that the ethnic and racial disparities are also considered as important factors that influence both the incidence and the efficacy of the treatment for this cancer [6]. In previous studies, it has been reported that there are differences in the clinical characteristics at diagnosis of Mexican patients with ALL in comparison to children from other populations where survival rates are better. For instance, almost 50% of ALL Mexican children are classified as having high risk of relapse according to the National Cancer Institute (NCI) criteria based on patient´s age and WBC count at diagnosis, whereas in developed countries, only one third of patients are classified as having a high risk of relapse using the same criteria [7, 8].

Furthermore, despite the use of the same chemotherapy regimens, in the last years it has been reported an upward trend since the incidence, relapses and mortality rates for Mexican children with ALL occur at a frequency of three times higher in comparison to that reported for children from developed countries [7]. Therefore, the identification of novel risk factors associated with ALL and the development of new therapeutic targets become extremely necessary for high risk populations.

As other cancers, ALL seems to be originated by multifactorial causes including both genetic and environmental. In the last decade, immune response has been demonstrated to play an essential role in the recognition and killing of cancer cells [9]. T lymphocytes along NK cells are immune cells that seem to dominate the immune response against leukemia [10]. Consequently, the important role of NK cells has been clearly demonstrated in the treatment of high risk leukemia [11]. Additional evidence has arisen from studies conducted in high-risk pediatric ALL patients undergoing hematopoietic stem cell transplantation (HSCT), showing the importance of selecting donors with allogenic NK cells to successfully cure patients [12, 13]. In contrast, an impaired NK cell-mediated cytotoxicity and/or abnormal expression of surface receptors has been observed in patients suffering from leukemia [14–16]. Notwithstanding, the studies have been conducted mostly in patients with chronic or myeloid leukemias [17–19]. A previous study has reported that peripheral blood NK cells from B-ALL patients have a compromised cytotoxicity towards K562 and autologous blasts. In addition, cell surface expression of NKp46 and NKG2A were altered in NK cells from B-ALL patients regarding age-matched healthy controls [20]. In the same study, Rouce and collaborators showed that after leukemia remission, NK cell-mediated cytotoxicity is recovered towards K562 but no towards autologous blast, indicating that blast undergo successful immune editing resulting in the selection of blast that avoid NK cell effector responses [20]. A recent investigation has suggested that a low NK cell production in bone marrow from patients suffering from ALL may contribute in disease progression. Moreover, an increase in the percentages of NK cells expressing class I restricted T-cell associated molecule (CRTAM) may be also associated with suppressor properties favoring malignant progression [21].

NK cells play an essential role in killing unwanted cells such as cancer and virus-infected cells. Unlike T and B lymphocytes, whereas antigen receptors dominate effector functions, NK cells express various germ-line encoding receptors that either activate or inhibit NK cell-mediated lysis of target cells [22]. A delicate balance between the signals emanating from activation and inhibition receptors dictate the outcome [23]. A major group of receptors influencing NK cell effector functions are those belonging to Killer cell immunoglobulin-like receptors (KIR) family. By recognizing MHC-I molecules on target cells, members of this family can either activate or inhibit NK cell function. Interestingly, an important association between a particular *KIR* genotype and an increased risk of leukemia has been reported [24]. Moreover, this association was more pronounced in Hispanic patients. Besides surface receptors, NK cells express an important number of signaling proteins including adapters and enzymes. Whereas genetic evidence has shown redundancy in signaling proteins governing NK cell-mediated effector functions [25], recent studies have led us to appreciate a unique role for a family of adapter proteins named signaling lymphocytic activation molecule (SLAM)-associated protein (SAP)-related adaptors [26, 27]. The SAP family of adapters are signaling proteins that play an essential role in dictate NK cell responsiveness [28, 29]. This family is composed by three members named SAP, EAT-2 (Ewing’s sarcoma-associated transcript 2) and ERT (EAT-2-related transducer, only in mouse). By mean of their SH2 domains, members of this family are able to interact with the immunoreceptor tyrosine-based switch motif (ITSMs) domains presents in the cytoplasmic region of the SLAM family of receptors [30]. Mouse models have shown that NK cells from mice lacking either SAP o simultaneously SAP, EAT-2 and ERT are unresponsive towards hematopoietic target cells whereas maintain responsiveness towards non-hematopoietic target cells [26]. These studies indicate the important role of SAP family proteins in governing NK cell-mediated cytotoxicity towards hematopoietic cells including malignancies. At present is unknown whether an altered expression of members of the SAP family in NK cells is associated with NK cell dysfunction favoring the emergence of hematological malignancies.

The aim of the present study was to perform a phenotypic, based on SAP expression, and a functional characterization, based on lysis of K562, of NK cells from children with high incidence for ALL at the moment of diagnosis and before treatment initiation.

## Material and methods

### Patients

The Mexican Inter-Institutional Group for identifying childhood leukemia causes (MIGICCL) conducted a case-control study. 41 cases were patients aged under 17 years diagnosed with ALL between July 1, 2016 and January 31, 2017, and treated in Mexico City public hospitals. Diagnosis of ALL was based on the morphologic and immunophenotypic features of leukemic cells. Peripheral blood samples (2–3 ml) from patients were obtained at the moment of diagnosis and before treatment initiation. 14 healthy controls were selected from the same health institution that referred the children with leukemia. The controls were children without leukemia matched with cases regarding age and sex. Children with the following diagnoses were not invited to participate: neoplasms, hematological diseases, allergies, infections, and congenital malformations. The main diagnoses of the controls were open fractures, hernias, orchidopexy, tonsillectomy, and other benign surgical diseases. Blood samples from the control group were taken at the time the patient was punctured before starting anesthesia and the surgical procedure.

### Clinical data collection and risk classification

Information regarding gender; age at diagnosis; white blood cell count (WBC); immunophenotype; dates of ALL diagnosis, treatment initiation, last visit, death, relapse, was collected from the patients’ clinical charts.

Risk classification at the moment of diagnosis was based on the National Cancer Institute [31] risk criteria. Patients between 1 and 10 years old and a leukocyte count <50 x 109/L were classified as NCI standard-risk whereas those aged ≥10 years or a leukocyte count ≥50 x 109/L were classified as NCI high-risk. All patients were treated according to the chemotherapy protocol used in the hospital where they received medical care.

Approval by National Scientific Research and Ethics Committee was obtained with the number R-2016-785-042. Furthermore, written informed consent was obtained from child´s parents and assent from patients ≥8 years of age.

### NK cell phenotyping

Peripheral blood mononuclear cells (PBMC) were isolated using Ficoll density separation (GE healthcare, Life Systems). For the evaluation of intracellular expression of SAP in NK cells, the PBMCs were stained with the following panel of fluorochrome-conjugated monoclonal Abs directed against cell surface markers: CD3 FITC (Biolegend, clone OKT3), CD56 APC (Biolegend, clone 5.1H11). Then, cells were fixed and permeabilized using Cytofix/Cytoperm Kit (BD Bioscience) and finally stained using the murine PE conjugated monoclonal antibody directed against human SAP (Thermo Scietific, clone XLP 1D12). An isotype control was used for every staining. Flow cytometric data were acquired on FACSCanto II (BD bioscience) and analyses with the use of FlowJo 7.6.5 software (Tree Star, Ashland, OR). Gates were set to exclude CD3^+^lymphocytes. Thereafter, NK cells were defined by the expression of CD56. The MFI SAP expression in NK cells was determined by using isotype control. SAP expression was analyzed in 18 B-ALL patients and 14 age-matched healthy controls.

### NK cell degranulation assays

NK cell degranulation assays were performed as previously described [32–34]. Briefly, PBMCs (1x10^6^/ml) were incubated with K562 cells (2x10^6^/ml) in a total volume of 200 ul in a 96 well plate. After 4 hours of incubation at 37°C, cells were recovered and stained using following antobodies: anti-CD3 FITC, anti-CD56 APC, and anti-CD107 PE (Biolegend, clone H4A3). GolgiStop was not included in these assays. Cells were acquired on FACSCanto II (BD bioscience) and analyses with the use of FlowJo 7.6.5 software (Tree Star, Ashland, OR). Gates were set to exclude CD3^+^ lymphocytes. Thereafter, the percentage of cells positive for CD107a was obtained after gating in CD3^-^CD56^+^ lymphocytes. The basal percentages for CD107a were obtained from PBMCs incubated alone. Degranulation was represented as ΔCD107a, which is the difference between the percentage of NK cells expressing surface CD107a after K562 stimulation and the percentage of NK cells expressing surface CD107a after incubation with medium alone. NK cell degranulation assays were performed in 41 acute leukemia patients and 14 age-matched healthy controls.

### Statistical analysis

Statistical analyses were performed by using SPSS IBM (Statistical Package for the Social Sciences, Inc., Version 21, Chicago, IL, USA). U-Mann Whitney Test was used to compare differences regarding phenotypic and functional features between cases and controls and disease subtypes and clinical subgroups (high vs. standard risk, WBC <50,000 and ≥50,000).

## Results

### Study population

Samples of 41 patients with ALL were included in the present analysis. The median age of patients was 11 years (range: 2–16 years). Of these, 35 corresponded to B-ALL, and 6 to T-ALL. There was a slight predominance of males (53.6%; n = 22). According to NCI risk classification criteria, 27 patients (65.8%) were high-risk patients. NK cell degranulation analysis was performed in a total of 41 ALL patients, whereas the assays for SAP expression and NK cell degranulation on same sample was possible to be conducted only in 18 patients. All data were compared with age-matched controls.

### Abnormal NK cell percentages in pediatric ALL patients

**Fig 1:**
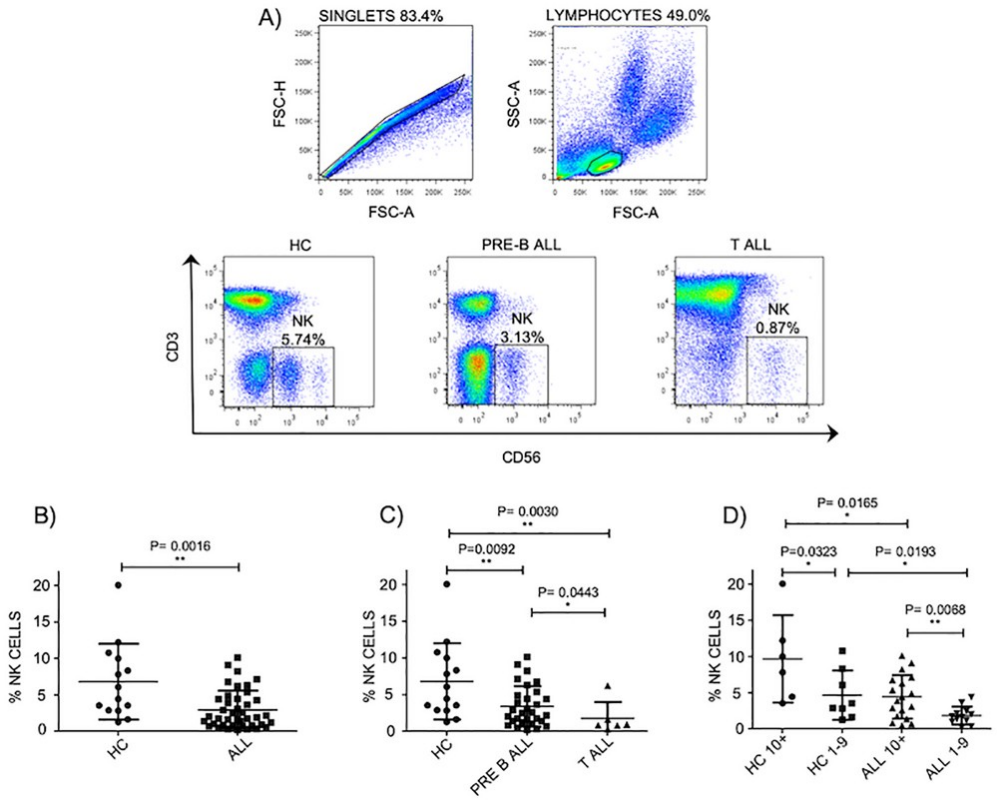
NK cell percentages are abnormal in pediatric ALL patients. A) Gating strategy to obtain NK cell percentages and representative FACS plots. NK cells from pediatric patients or healthy controls were defined by gating on the CD3-CD56+ lymphocyte fraction of PBMCs. B) The Percentages of NK cells in the lymphocyte gates were analyzed in 41 patients with ALL at moment of diagnosis compared to 14 healthy age-matched controls. C) Percentages of NK cells were analyzed according to immunophenotype in 30 pre-B-ALL patients and 6 T-All patients. D) Percentages of NK cells were analyzed according to age in 30 pre-B-ALL patients compared to 14 age-matched controls. Error bars denote standard error of the mean between individuals within group. P values report significance according to U-Mann Whitney Test one-tail. All data were acquired on FACSCanto II (BD bioscience) and analyses with the use of FlowJo 7.6.5 software (Tree Star, Ashland, OR).

It has been reported that patients suffering from leukemia have abnormal percentages and absolute numbers of NK cells at moment of diagnosis [35, 36]. Therefore, we decide to evaluate the percentages of NK cells in pediatric patients suffering from ALL. In our study, at moment of diagnosis, we found a significant difference in the percentages of NK cells in ALL patients compared to age-matched controls as previously reported (Fig 1A and 1B). Moreover, a significant difference was also observed in pre-B-ALL patients compared to T-ALL patients, where those with T-ALL displayed the lowest percentages (Fig 1C). Interestingly, when ALL patients were stratified according to age as risk factor, the percentages of NK cells in ALL patients between 1 and 9 years of age were significantly lower compared to ALL patients aged 10 years or older (Fig 1D). These data suggest that the percentages of peripheral blood NK cells are lower in ALL patients.

### NK cell-mediated cytotoxicity is impaired in patients with ALL

**Fig 2:**
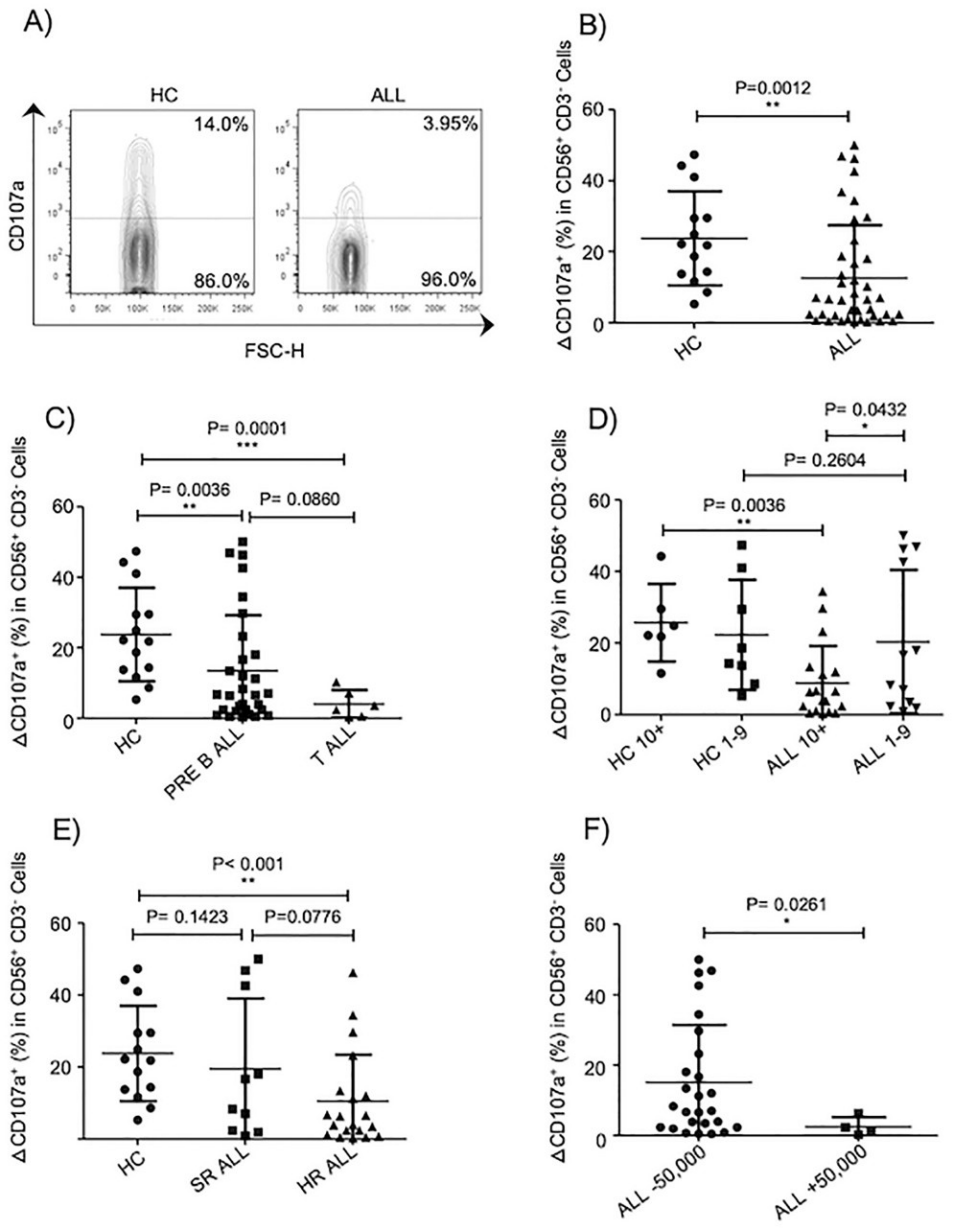
NK cell-mediated cytotoxicity is impaired in patients with ALL. A) Representative FACS plots depict NK cell response (CD107a degranulation) against K562 cells. B) NK cell degranulation assays were performed in 41 acute leukemia patients and 14 healthy age-matched controls. C) NK cell degranulation assays were performed in 30 pre-B-ALL, 6 T-ALL patients and 14 age-matched controls. D) NK cell degranulation was analyzed according to age in 30 pre-B-ALL patients and 14 age-matched controls. E) NK cell degranulation was analyzed according to high risk or standard risk in 30 pre-B-ALL patients and 14 age-matched controls. F) NK cell degranulation was analyzed according to WBC in 30 pre-B-ALL patients and 14 age-matched controls. Degranulation was represented as ΔCD107a, which is the difference between the percentage of NK cells expressing surface CD107a after K562 stimulation and the percentage of NK cells expressing surface CD107a after incubation with medium alone. Error bars denote standard error of the mean between individuals within group. P values report significance according to U-Mann Whitney Test one-tail.

An impaired NK cell effector functions has been associated with a higher incidence of cancer including hematological malignancies [37, 38]. There are few studies assessing the functional competence of NK cells in pediatric patients with ALL. The observation by Rouce and collaborators has provided information on the state of NK cell responsiveness at moment of diagnosis and remission [20]. However, none study has provided information on whether NK cell-mediated cytotoxicity results equally affected taking in consideration important prognostic features and risk factors. Our data showed, as evidenced by degranulation assays using K562 as target cells, that peripheral blood NK cells from ALL patients have an impaired NK cell cytotoxicity compared to age matched-controls (Fig 2A and 2B). The impaired NK cell-mediated lysis of K562 was more significantly affected in those patients suffering from T-ALL compared to B-ALL (Fig 2C). Interestingly, those pediatric ALL patients with ages above 10 years showed a significant lower NK cell degranulation compared to patients with ages between 1- and 9-year-old (Fig 2D). Moreover, in those patients stratified into a high-risk population, the NK cell degranulation was significantly reduced compared to standard-risk population to compared to age-matched controls (Fig 2E). Interestingly, NK cell cytotoxicity was compromised in those B-ALL patients with WBC over 50,000xmm3 compared to B-ALL patients with WBC below 50,000xmm3 (Fig 2F). These data suggest that NK cell cytotoxicity is not equally affected in all pediatric patients suffering with ALL.

### SAP expression in NK cells from B-ALL patients is partially reduced

**Fig 3:**
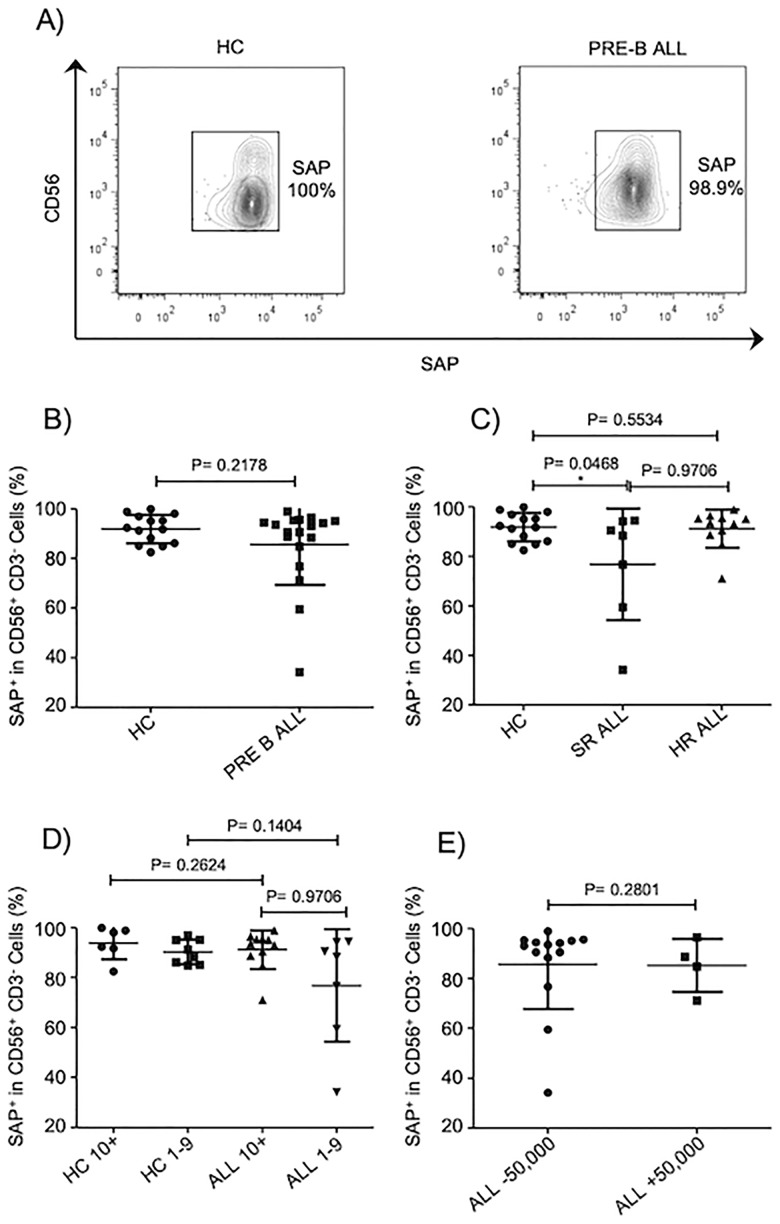
Frequencies of NK cells expressing SAP are normal in patients with B-ALL. A) Representative FACS plots depict frequencies of SAP+ NK cells. B) Frequencies of CD3^-^CD56^+^ cells expressing SAP were analyzed in 18 pre-B-ALL patients compared to 14 age-matched controls. C) Frequencies of CD3-CD56+ cells expressing SAP were analyzed according to risk factor in 18 pre-B-ALL patients and 14 age-matched controls. D) Frequencies of CD3^-^CD56^+^ cells expressing SAP were analyzed according to age in 18 pre-B-ALL patients and 14 age-matched controls. E) Frequencies of CD3^-^CD56^+^ cells expressing SAP were analyzed according to WBC in 18 pre-B-ALL patients and 14 age-matched controls. Error bars denote standard error of the mean between individuals within group. P values report significance according to U-Mann Whitney Test one-tail.

SAP has been reported to be critical in regulating NK cell responsiveness towards hematopoietic cells [26, 29]. Various mouse models indicate that NK cells lacking SAP expression loss the ability to kill hematopoietic cells whereas retain their competence to eliminate non-hematopoietic cells. In humans, SAP deficiency as consequence of deleterious mutations in *SH2D1A* results in an immunodeficiency characterized by an abnormal susceptibility to EBV infections. As consequence, lymphoproliferative disorders are observed in these patients probably caused by inability of NK cells to properly eliminate transformed cells and/or antigen presenting cells. Despite the importance of SAP in regulating NK cells effector functions, no other human pathology has been associated with SAP abnormal expression or function. As SAP plays a critical role in regulate NK cell responsiveness towards hematopoietic cells, we decided to evaluate whether SAP expression is abnormal in NK cells from patients suffering ALL leukemia at moment of diagnosis. Our data indicate that there is not a significant difference in the frequencies of NK cells expressing SAP between B-ALL patients and age-matched controls (Fig 3A and 3B). Moreover, the frequencies of NK cells expressing SAP remain normal when patients were stratified according to various prognostic features and risk factors (Fig 3C–3E). Although we did observe a significant difference only in patients with standard-risk compared to healthy controls, we did not observe any difference when healthy donors were compared to those ALL patients in high-risk group. These results suggest that the frequencies of NK cells expressing SAP remain unaffected in patients with B-ALL.

**Fig 4:**
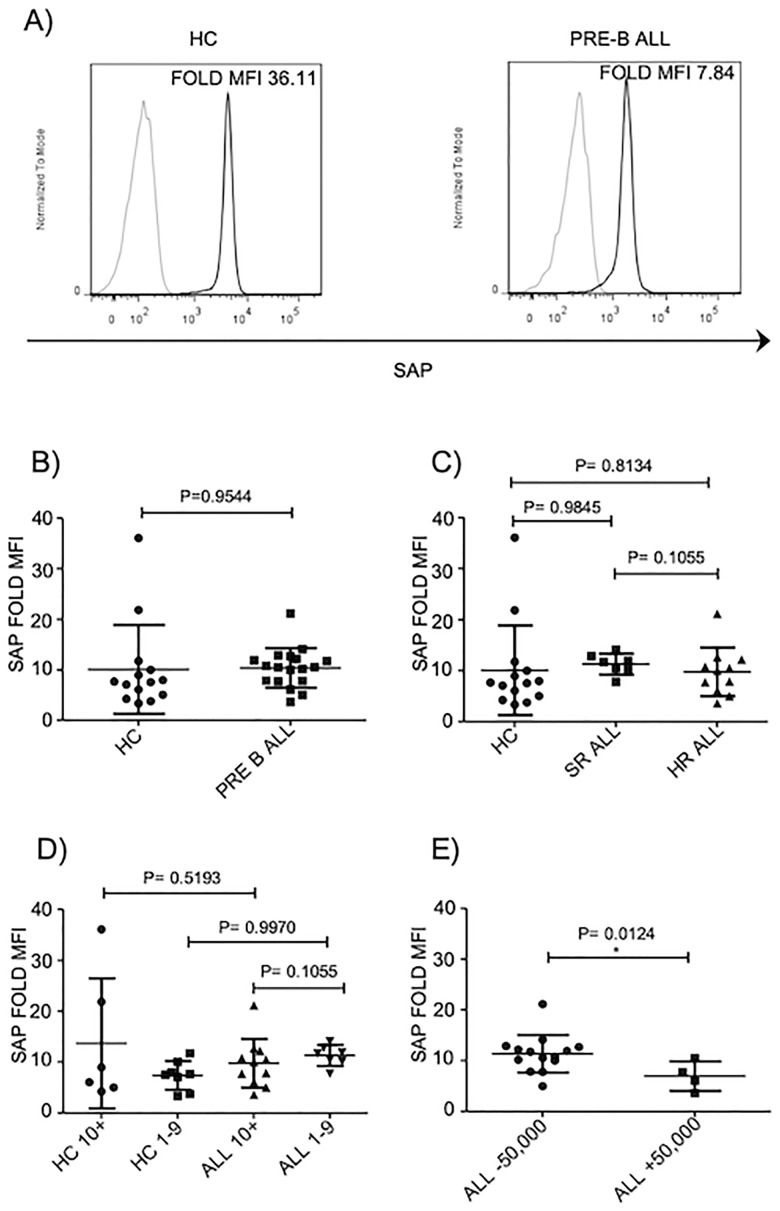
SAP expression is partially reduced in NK cells from B-ALL patients. A) Representative FACS histogram depict MFI of SAP+ NK cells. B) SAP fold MFI for CD3-CD56+ cells was analyzed in 18 pre-B-ALL patients regarding 14 age-matched controls. C) SAP fold MFI for CD3-CD56+ cells was analyzed according to risk factor in 18 pre-B-ALL patients and 14 age-matched controls. D) SAP fold MFI for CD3-CD56+ cells was analyzed according to age of 18 pre-B-ALL patients and 14 age-matched controls. E) SAP fold MFI for CD3-CD56+ cells was analyzed according to WBC in 18 pre-B-ALL patients. Error bars denote standard error of the mean between individuals within group. P values report significance according to U-Mann Whitney Test one-tail.

In addition to frequencies of NK cells expressing SAP, we decide to evaluate the mean fluorescence intensity (MFI) of SAP in NK cells. We did not find a significant difference in SAP expression in NK cells from healthy controls and pre-B ALL patients (Fig 4A and 4B) Moreover, when patients were stratified according to other prognostic factors (age or risk factor) none difference was found between patients and age-matched healthy controls (Fig 4C and 4D). In contrast, we found that NK cells from patients with WBC over 50,000xmm^3^ express less SAP compared to patients with WBC below 50,000xmm^3^ (Fig 4E).

### A positive correlation between SAP expression and a reduced NK cell cytotoxicity is revealed in B-ALL patients with WBC over 50,000xmm^3^

**Fig 5:**
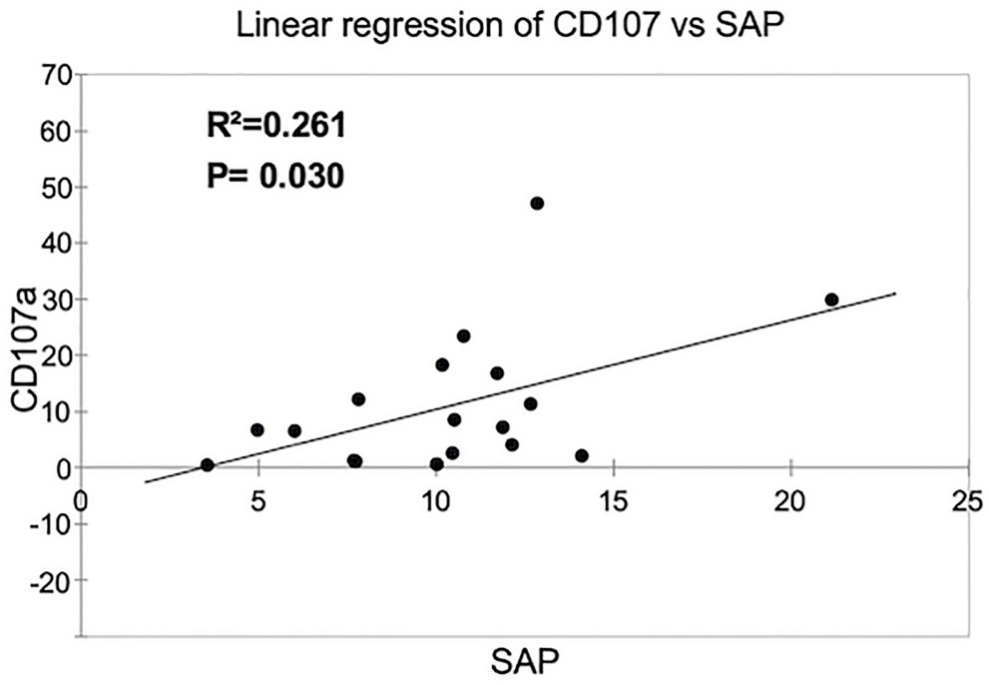
A positive correlation between SAP expression and a reduced NK cell cytotoxicity is revealed in B-ALL patients with WBC over 50,000xmm^3^. A positive correlation study was applied to 18 pre-B-ALL patients, where was possible to determine both NK cell-mediated cytotoxicity and SAP analysis expression. The R^2^- and P values are indicated.

A reduction or absence of SAP expression results in a significant reduction in the ability of NK cells to kill malignant hematopoietic cells as evidenced in NK cells from patients with XLP1 [29, 39, 40]. Therefore, we determined whether a reduction in the expression of SAP observed in those patients with WBC over 50,000 correlates with an impaired NK cell-mediated cytotoxicity. By using a lineal regression analysis, our data demonstrated a positive correlation between a low expression of SAP and a reduced NK cell cytotoxicity in pediatric patients suffering from B-ALL (Fig 5).

## Discussion

Acute lymphoblastic leukemia represents the most common and lethal cancer during childhood [41, 42]. Whereas genetic and environmental factors are important factors that predispose the outcome, immune cells are now being recognized as determinants in the development of leukemia and other cancers. Among immune cells, NK cells play an important role in recognizing and killing leukemia cells [22, 38]. In consequence, NK cell-based immunotherapies have proven to be successful in the treatment of high-risk leukemia [38, 43]. However, it is also noticed that not all leukemias, either myeloid or lymphoid, are equally susceptible to these cell-based therapy regimens. Therefore, a phenotypic and functional characterization of NK cells under specific leukemia scenarios become essential in order to understand the particularities related to NK cells and different kinds of leukemia.

In this work, we presented a phenotypical and functional characterization of peripheral blood NK cells from pediatric patients suffering from ALL at moment of diagnosis. As shown by previous studies, we did observe a significant difference in the percentages of peripheral blood NK cells from patients at moment of diagnosis compared to healthy age-matched controls [35, 36]. This was also true when NK cell percentages were analyzed taking into consideration prognostic features such as WBC and whether leukemia was from T or B cell-type. Notably, a significant reduction in percentages of NK cells was also noticed when patients were stratified according to age, whereby those aged between 1 to 9 years display lower percentages of NK cells compared to patients aged 10 years old and above. As far as we know, our study is the first study conducted in patients from Mexico City, which have one of the highest risk factors for developing ALL worldwide.

Similar to results obtained by Rouce and colleagues, NK cell-mediated cytotoxicity towards K562 cells was reduced in peripheral blood NK cells from ALL leukemia patients compared to age-matches controls. This impaired NK cell-mediated cytotoxicity was observed in patients with both B-ALL and T-ALL. Interestingly, when NK cell-mediated lysis towards K562 was analyzed taking into consideration different prognostic features, an impaired NK cell-mediated cytotoxicity was observed only in patients classified as high- but not as low-risk compared to age-matched healthy controls. Interestingly, a lower NK cell-mediated cytotoxicity was also noticed in ALL patients aged over 10 years old and above compared to both healthy controls of same age, and to ALL patients aged between 1–9 years old. In contrast, ALL patients in the 1-9-year cohort did not display a lower NK cell-mediated cytotoxicity compared to age-matched healthy controls. Finally, an impaired NK cell-mediated cytotoxicity was also observed in those B-ALL patients with WBC over 50,000xmm3 compared to B-ALL patients with WBC below 50,000xmm3. These data suggest that an abnormal NK cell effector function is not necessarily equally observed in all pediatric B-ALL patients and that there are other factors that may contribute to determine whether the NK cell-mediated cytotoxicity will result impaired or not. According to our data, the WBC and patients age, resulted to be the major factors that were associated with an abnormal NK cell-mediated cytotoxicity. Interestingly, both WBC over 50,000xmm3 and age, are two reliable prognostic factors used to determine the risk factor and the probability of remission [2, 44]. Based on these findings, our study suggest that an impaired NK cell mediated cytotoxicity can also emerge as a possible prognostic factor for high risk pediatric patients suffering from B-ALL, complementing observations made by Agnieszka et al [45].

Minimal residual disease (MDR) has recently been describe as a reliable factor to determine risk factor and the probability of remission for ALL patients. In our study, we lack any information regarding the status for MRD in our patients, however it will be interesting to evaluate in further studies a possible association between an abnormal NK cell-mediated cytotoxicity and MRD. In addition, in only 17.7% of ALL patients from Mexico City, the four most common gene rearrangements have been detected [14, 46], making difficult to address how NK cell function may be affected by such genetic alterations in patients with ALL. With no doubt, this remain an interesting issue to be addressed in future studies.

The finding that those patients with WBC over 50,000xmm3 displayed a reduced NK cell lysis towards K562 at moment of diagnosis, suggest an important association between a compromised NK cell effector function and high probability of re-incidence of the disease. However, at this point, it is not clear why not all B-ALL patients displayed a significant reduction in the ability of NK cells to kill target cells and further studies will be necessary to address this issue.

SAP family adapters play an essential role in regulate NK cells lysis towards hematopoietic cells. Individuals carrying missense mutations in the gene encoding for SAP, develop a lymphoproliferative syndrome as consequence of an abnormal susceptibility of EBV infections [47, 48] and very often, these individuals develop lymphoma. Besides XLP-1, there are no other evidences where an abnormal SAP expression or function is associated with a human pathology. As far as we know, our study is the first evidence that in those pediatric B-ALL patients with WBC over 50,000xmm3, the NK cells display a partial decrease in SAP expression regarding those patients with WBC below 50,000xmm3. Moreover, by using a lineal regression analysis, our data demonstrated a positive correlation between a low expression of SAP and a reduced NK cell cytotoxicity in pediatric patients suffering from B-ALL. Therefore, our data suggest that a partial decrease in SAP expression may contribute with an impaired NK cell-mediated cytotoxicity observed in B-ALL patients. Our study also provide evidence that an abnormal NK cell phenotype and function are not equally displayed in pediatric patients suffering from B-ALL. These data suggest that there are unidentified factors that might contribute to influence NK cell biology in patients with leukemia. In contrast, NK cell mediated-cytotoxicity and SAP expression become important variables that might be useful to determine the patient outcome.

## Conclusions

In the present study it was observed that NK cells from children with ALL harbor an impaired cytotoxicity towards K562 at moment of diagnosis. A more detailed analysis taking into consideration prognostic features and risk factor, revealed that NK cell phenotype and function are not equally affected in all B-ALL patients. Moreover, NK cell-mediated cytotoxicity was compromised in those patients with a leukocyte count ≥50,000xmm3 where also it was found a decreased expression of SAP regarding patients with a leukocyte count < 50,000xmm3. These data suggest a positive correlation between low SAP expression and decreased NK cell-mediated cytotoxicity in pediatric patients suffering from ALL.
