# Supplementary material for: Functional characterization of NK cells in Mexican pediatric patients with acute lymphoblastic leukemia: Report from the Mexican Interinstitutional Group for the Identification of the Causes of Childhood Leukemia
Source: PLoS One. 2020 Jan 17;15(1):e0227314. doi: 10.1371/journal.pone.0227314 (PMC6968843; doi:10.1371/journal.pone.0227314)
Supplement: Attachment — Submitted filename: Response to Reviewers.docx [file pone.0227314.s001.docx]

November 15, 2017

Professor Joerg Heber

Editor-in-Chief

*Plos One*

Dear Dr. Heber:

You will find enclosed the revised version of manuscript PONE-D-19-20648, which we would like considered for publication in **PlosOne**.

We are pleased that the Editors and Reviewers were very supportive of our manuscript. As requested, we have addressed the queries of the Reviewers, by adding additional data and clarifications in the text. The corresponding changes in the text are highlighted in yellow. As a result of these various changes, we believe that the manuscript is much improved. We have also modified the manuscript according to the PlosOne format instructions and publication policies.

Below is a point-by-point response to the Reviewers’ comments.

**Major Points**

**Reviewer #1:**

*1. Enlarge the number of healthy donors and patients.*

We agree with reviewer that the number of controls and patients is unbalanced. In order to improve the number of healthy controls analyzed, we have included 6 more age-matched healthy controls to the study. However, it is important to mention that statistical conclusions are possible taking into account that with the sample size analyzed of cases and controls, the differences observed were statistically significant with an adequate statistical power. Considering the sample size here analyzed, if the pooled variance would have been so small as 5 or so high as 20; therefore, the sample size used has a power to detect differences of 5% among the groups with a power of 94.38%. If the difference between the two groups would have been 10% or higher, the power to detect differences is of 100%. In the case of analyses of subgroups with 8 patients, differences of 5% and a pooled variance of 5% too, the statistical power was of 98.8%. For differences of 7% among subgroups, the power was of 100%. When we compared subgroups of 5 patients with differences of 5% among groups and the pooled variance was 5% too, the power was of 89.75%. When a differences of 10% was observed, the statistical power was of 100%.

On the other hand, it is important to mention that a lower number of controls is not an impediment for made comparisons between cases and controls. Although we agree that the inclusion of more controls could enhance the statistical power, the associations remained statistically significant after the inclusion of more controls.

*2. For the cytotoxic and degranulation assays, at least three serial dilutions have to be performed to evaluate NK activity. We suggest aliquots of 100 μl from each NK cell serial dilution containing 2 x 105, 1 x 105 and 0.5 x 105 cells, in triplicates, and aliquots of 100 μl of target cells (1 x 105cells/well) to be added to generate 2:1, 1:1 and 0.5:1 effector—to-target ratio (E:T ratio). In fact, cytotoxicity is more effective at higher E/T ratios, while degranulation responses are stronger at low E/T ratios.*

We concur with the Reviewer that cytotoxic assays are also informative in evaluating NK cell function, however the paucity of peripheral blood available from each patient it makes difficult to performed both assays (specific lysis and degranulation assays) in all samples. Moreover, degranulation assays is a well-known standard method that allow to assess NK cell-mediated cytotoxicity and this method is broadly used not only in basic approaches to assess NK cell function but also for diagnosis of pathologies related to an abnormal NK cell function. We have included the proper references in Materials and methods.

*3. Together with the frequency (percentage) of NK cells, please report their absolute number.*

We agree with reviewer that this is an important information, unfortunately this information was not available for all samples.

*4. In Figure 1B, indicate the p value comparing ALL 10+ and ALL 1-9 that could be the most interesting results you obtained. Eliminate HD or increase their number. Eliminate the comparison between ALL <50000 and >50000 for the same reason.*

The requested p value has been indicated in revised Figure 1. In addition, we have increased the number of HC as suggested. The comparison between ALL <50000 and >50000 has been eliminated in the new figure 1 as requested.

*5. Why in figure 2C there is less ALL 1-9 than in figure 1? It could be interesting to understand if they are less numerous compare to ALL 10+ (Fig1B) but more functional.*

This is an excellent point. This information has been corrected accordingly. These data are mentioned on p. 13, lines 298-300 and p. 15, lines 325-327

*6. Since the paper seeks to clarify why in the Mexican population there is a higher frequency of high-risk relapsing patients and find new risk factors, according to this reviewer opinion, a gene expression analysis, or at least the analysis of NKG2D ligands, could improve the findings of this work and let to more interesting conclusions.*

We agree that a gene expression analysis, or at least the analysis of NKG2D ligands, could improve the findings of this work, however we considered that the gene expression analysis was not the scope of this manuscript or it will be part of other manuscript.

**Minor Points**

*7. Elimination of leukemia cells relies mainly on their recognition by both T cells and NK cells. Please revise the statement “NK cells are unique lymphocytes that seem to dominate the immune response against leukemia”, otherwise justify it with proper citations from literature.*

We agree with the reviewer; the statement has been modified accordingly throughout the text.

*8. Please specify the type of samples collected and the number of cases analyzed (healthy age-matched controls and patients) in the Abstract and in the “Material and Methods” paragraph.*

We have included this information in the revised manuscript as requested by the reviewer. This information is mentioned on p. 4, lines 83-84, and p.4, lines 87-88.

*9. To estimate the degranulation of NK cells using anti-CD107a mAb, GolgiStop has to be added. Did you? Please clarify the protocol used.*

We agree with the reviewer that the addition of GolgiStop is included in some protocols, however there are other that not include the use of GolgiStop, as is in this case. This information has been clarified in Materials and Methods. In addition, the proper references have been included.

*10. SAP evaluation in NK cells is innovative. However, according to this reviewer opinion, to see differences among the groups analyzed, the analysis has to be revised indicating the frequency of SAP expressing NK cells and not the expression of SAP inside the NK cell gate.*

This point was included in the analysis as suggested by reviewer.

*11. In the figure 1 legend, correct CD65 with CD56.*

This was corrected in the text as indicated.

*12. Are they only B-ALL patients in figure 3 and 4 as described in the text?*

For figures 3 and 4 only B-ALL patients were included as indicated in the text.

*13. - Why adverse cytogenetic abnormalities have not been considered as risk factors?*

This is an excellent point; however, gene rearrangements are found in only 17.7% of the patients with acute lymphoblastic leukemia from Mexico City. This information has been included in the discussion to clarified the point indicated by the reviewer. p. 21, lines 466-469.

*14. Please better describe the FACS gating strategy. Pay attention to define NK cells as CD56+ cells among CD3- lymphocytes. Specify if and how you discriminate between progenitors and mature lymphocytes*.

We have better described these points in the revised manuscript as indicated by the reviewer. Since all analysis were performed in mononuclear cells form peripheral blood, we did not discriminate between progenitors and mature lymphocytes.

*15. Please show the gating strategy and the representative FACS dot plots/histograms in each figure.*

A representative FACS plots/histogram has been included in each figure as suggested by the reviewer.

*16. Figure 1 A. Since T ALL are underrepresented compared to B ALL, I suggest not to separate B and T ALL.*

This is an important suggestion, however we think that data is important as this information has not been published previously.

*17. In figure 2, correct the legend of the Y-axes: CD107a+ in CD56+CD3- cells. Calculate and report also the number of CD107a+ NK.*

The legend has been corrected as suggested by the reviewer. Unfortunately, we did not analyze the absolute number of NK cells in this study or this information was not available for all samples.

*18. In figure 3, correct the legend of the Y-axes: SAP+ in CD56+CD3- (NK) cells. Calculate also the number of SAP+ NK that could be more informative.*

The legend has been corrected as suggested by the reviewer. Unfortunately, we did not analyze the absolute number of NK cells in this study, or this information was not available for all samples.

*19. Figure 5. The statistic is missing.*

The statistic has been incorporated in corresponding figure as indicated by reviewer.

**Reviewer #2:**

*1. In the introduction you can also comment on the study by de Smith et al in Blood (2014) where they found a correlation between a KIR A haplotype and leukemia risk in hispanic children, this is very relevant information for the current study.*

This is an excellent point. The study referred by the reviewer has been commented in the introduction as indicated in p. 8, lines 173-175.

*2. Materials and methods: Please state the volume of blood drawn, and also include in this section information on the number of patients and healthy controls (even though this is stated in the Results section, it should also be included here).*

We have included this information as requested by reviewer.

3. Please provide the supplier of the antibodies used in this study.

This information has been included in Materials and Methods.

4. Please explain what is meant by "fold MFI"; is it MFI of SAP in stained samples vs isotype controls? Also MFI is several places misspelled as MIF.

This information has been corrected.

5. For degranulation, please clarify how deltaCD107a is calculated; is it the difference between CD107a measured against targets vs NK cells without targets?

This information has been clarified in Materials and Methods. See p. 12, lines 265-268

6. Also clarify in the materials and methods the gating strategy used. For instance on line 255, it is written that a gate was set on CD3+ lymphocytes, did the authors mean to say "CD3- lymphocytes"? I assume that the authors have gated cells as CD3-CD56+ throughout the manuscript?

We appreciate this comment from reviewer. This point has been clarified in the Materials and Methods section.

7. In the results section, the text describing panel 2C and 2D has been mixed up.

This point has been corrected accordingly.

8. The figure describing impact of age on NK cell degranulation is missing several data points, please explain why or add the missing data points.

This point has been corrected.

9. The authors report that high WBC translate into poor degranulation; can the authors discuss whether this also can be the by lower numbers of NK cells in the PBMC sample that can result in sub-optimal assay?

This is an excellent point by the reviewer. Whereas this possibility can not be formally excluded, we did not find a significant difference in the percentages of NK cells between patients with high or low WBC.

10. I suggest to merge figure 3 and 4 into one figure, and take out the graphs showing boys/girls as there is no difference between different sexes. Also write on the y-axis "%SAP+ NK cells", instead of "% NK SAP+".

We appreciate the suggestions by the reviewer. The y-axis has been corrected, and graphs showing boys/girls has been eliminated. Merge of figures 3 and 4 was not possible since new data was incorporated as suggested by reviewer #1.

11. In Figure 3 and 4 there are 18 data points, not 35 data points as stated in the figure legends, please modify.

The legend has been modified accordingly.

12. Figure legends can be shortened, do not need to repeat methods, they are already in the materials and methods section.

Figures legends were shortened as suggested by reviewer.

13. The text for Figure 4 does not match the actual figure, please modify.

The Figure 4 and text has been corrected.

14. Figure 5: Please provide r, and the p-value.

These values have been incorporated in figure 5.

15. There are many grammatical and typographical errors in the manuscript, and I suggest that the authors critically re-read the text and correct these. For instance, write "compared to" instead of "regarding".

We appreciate the comments by the reviewer. Grammatical and typographical errors have been revised and corrected.
