# Supplementary material for: Functional characterization of NK cells in Mexican pediatric patients with acute lymphoblastic leukemia: Report from the Mexican Interinstitutional Group for the Identification of the Causes of Childhood Leukemia
Source: PLoS One. 2020 Jan 17;15(1):e0227314. doi: 10.1371/journal.pone.0227314 (PMC6968843; doi:10.1371/journal.pone.0227314)
Supplement: Attachment — Submitted filename: Response to Reviewers.docx [file pone.0227314.s002.docx]

December 5, 2019

Professor Joerg Heber

Editor-in-Chief

*Plos One*

Dear Dr. Heber:

You will find enclosed the revised version of manuscript PONE-D-19-20648, which we would like considered for publication in **Plos One**.

We are very pleased that the Editors and Reviewers were very supportive of our manuscript. As requested, we have addressed the queries of the Reviewer #1, by doing clarifications in the text. The corresponding changes in the text are highlighted in yellow. As a result of these changes, we believe that the manuscript is improved. We have also modified the manuscript according to the PlosOne format instructions and publication policies.

Below is a point-by-point response to the comments of the Reviewer #1.

**Reviewer #1:**

**Minor suggestions**

*1. line 302 please use "lower" instead of "abnormal"*

We have use lower instead of abnormal in the corresponding line.

*2. line 305 percentages of nk cell in the lymphocyte gates.*

We have included in the text the suggestion made by the reviewer.

*3. Fig 2 c, d, e. You can appreciate two clusters. Please be sure that data are correct and not due to acquisition byass. Same day of acquisition?*

This is an interesting point. We agree with the reviewer that two cluster can be observed in some graphics, however we have checked all data and we are certainly that information is correct and no due to acquisition artefacts. We did not acquire all samples the same day. To ensure the reliability of our results for immunophenotyping and functional assays, all experiments were analyzed in a flow cytometer FACS ARIA III (BD) with a biannual maintenance, inspection and correction service. Additionally, the quality of measures is sustained by daily quality controls monitoring overall flow cytometric performance (laser alignment and fluorescence stability).

**Major suggestions**

*4. line 368 "significant difference" it is right but p value is 0.0468. Please pay attention to not overestimate data and conclusions.*

We agree with the reviewer. We have put attention in order to not overestimate data and conclusions by modifying the respective phrase in the text.

“Although we did observe a significant difference only in patients with standard-risk compared to healthy controls, we did not observe any difference when healthy donors were compared to those ALL patients in high-risk group…”

*5. line 450 "severe impaired nk cytotoxicity" is not a correct definition. p value is 0.02 and sample size is little and this could bring to uncertain conclusions. The same at line 499, please eliminate "severely".*

We agree with the reviewer. We have made the suggestions made by the reviewer by not using the word “severe” or “severely” throughout the text.

“Moreover, patients presenting a leukocyte count ≥ 50,000xmm^3^ displayed a ~~severe~~ reduction in NK-cell mediated cytotoxicity….”

“In addition, NK cell function was observed to be ~~severely~~ compromised in patients with a leukocyte count….”

“Interestingly, NK cell cytotoxicity was ~~severely~~ compromised in those B-ALL patients with WBC over….”

“Finally, a ~~severe~~ impaired NK cell-mediated cytotoxicity was also observed in those B-ALL patients with WBC over 50,000xmm3 compared to B-ALL patients with WBC below 50,000xmm3….”

“The finding that those patients with WBC over 50,000xmm3 displayed a ~~severely~~ reduced NK cell lysis towards K562….”

“Moreover, NK cell-mediated cytotoxicity was ~~severely~~ compromised in those patients with a leukocyte count ≥50,000xmm3 where also it was found a decreased expression of SAP regarding patients with a leukocyte count < 50,000xmm3…”

We thank you and the Reviewers for your support and constructive comments. We hope that our revised in now suitable for publication in **Plos One**.

Sincerely,

Mario E. Cruz-Muñoz

Juan Manuel Mejía-Aranguré
