# Supplementary material for: Functional characterization of NK cells in Mexican pediatric patients with acute lymphoblastic leukemia: Report from the Mexican Interinstitutional Group for the Identification of the Causes of Childhood Leukemia
Source: PLoS One. 2020 Jan 17;15(1):e0227314. doi: 10.1371/journal.pone.0227314 (PMC6968843; doi:10.1371/journal.pone.0227314)
Supplement: Peer review history

# Peer review: Decision Letter 0

**DOI:** 10.1371/journal.pone.0227314.r001

**Authors:** Francesco Bertolini

4 Oct 2019

PONE-D-19-20648

Functional characterization of NK cells in Mexican pediatric patients with acute lymphoblastic leukemia: Report from the Mexican Interinstitutional Group for the Identification of the Causes of Childhood Leukemia.

PLOS ONE

Dear Dr Cruz-Munoz,

Thank you for submitting your manuscript to PLOS ONE. After careful consideration, we feel that it has merit but does not fully meet PLOS ONE’s publication criteria as it currently stands. Therefore, we invite you to submit a revised version of the manuscript that addresses the points raised during the review process by both reviewers, experts in the field.

We would appreciate receiving your revised manuscript by Nov 18 2019 11:59PM. When you are ready to submit your revision, log on to https://www.editorialmanager.com/pone/ and select the 'Submissions Needing Revision' folder to locate your manuscript file.

If you would like to make changes to your financial disclosure, please include your updated statement in your cover letter.

To enhance the reproducibility of your results, we recommend that if applicable you deposit your laboratory protocols in protocols.io, where a protocol can be assigned its own identifier (DOI) such that it can be cited independently in the future. For instructions see: http://journals.plos.org/plosone/s/submission-guidelines#loc-laboratory-protocols

Please include the following items when submitting your revised manuscript:

- A rebuttal letter that responds to each point raised by the academic editor and reviewer(s). This letter should be uploaded as separate file and labeled 'Response to Reviewers'.
- A marked-up copy of your manuscript that highlights changes made to the original version. This file should be uploaded as separate file and labeled 'Revised Manuscript with Track Changes'.
- An unmarked version of your revised paper without tracked changes. This file should be uploaded as separate file and labeled 'Manuscript'.

Please note while forming your response, if your article is accepted, you may have the opportunity to make the peer review history publicly available. The record will include editor decision letters (with reviews) and your responses to reviewer comments. If eligible, we will contact you to opt in or out.

We look forward to receiving your revised manuscript.

Kind regards,

Francesco Bertolini, MD, PhD

Academic Editor

PLOS ONE

Journal Requirements:

When submitting your revision, we need you to address these additional requirements.

1. Please ensure that your manuscript meets PLOS ONE's style requirements, including those for file naming. The PLOS ONE style templates can be found at http://www.journals.plos.org/plosone/s/file?id=wjVg/PLOSOne_formatting_sample_main_body.pdf and http://www.journals.plos.org/plosone/s/file?id=ba62/PLOSOne_formatting_sample_title_authors_affiliations.pdf

Additional Editor Comments (if provided):

[Note: HTML markup is below. Please do not edit.]

Reviewers' comments:

Reviewer's Responses to Questions

**Comments to the Author**

1. Is the manuscript technically sound, and do the data support the conclusions?

The manuscript must describe a technically sound piece of scientific research with data that supports the conclusions. Experiments must have been conducted rigorously, with appropriate controls, replication, and sample sizes. The conclusions must be drawn appropriately based on the data presented. 

Reviewer #1: No

Reviewer #2: Partly

**********

2. Has the statistical analysis been performed appropriately and rigorously? 

Reviewer #1: No

Reviewer #2: Yes

**********

3. Have the authors made all data underlying the findings in their manuscript fully available?

The PLOS Data policy requires authors to make all data underlying the findings described in their manuscript fully available without restriction, with rare exception (please refer to the Data Availability Statement in the manuscript PDF file). The data should be provided as part of the manuscript or its supporting information, or deposited to a public repository. For example, in addition to summary statistics, the data points behind means, medians and variance measures should be available. If there are restrictions on publicly sharing data—e.g. participant privacy or use of data from a third party—those must be specified.

Reviewer #1: Yes

Reviewer #2: Yes

**********

4. Is the manuscript presented in an intelligible fashion and written in standard English?

PLOS ONE does not copyedit accepted manuscripts, so the language in submitted articles must be clear, correct, and unambiguous. Any typographical or grammatical errors should be corrected at revision, so please note any specific errors here.

Reviewer #1: Yes

Reviewer #2: No

**********

5. Review Comments to the Author

Please use the space provided to explain your answers to the questions above. You may also include additional comments for the author, including concerns about dual publication, research ethics, or publication ethics. (Please upload your review as an attachment if it exceeds 20,000 characters)

Reviewer #1: The presence of NK cells in the bone marrow at diagnosis can be a prognostic factor in children with ALL (doi: 10.1155/2019/3596983). In the submitted work, Valenzuela-Vazquez and collogues proposed a phenotypical and functional characterization of NK cells in children with ALL comparing them to age-matched healthy donors. According to the opinion of this reviewer, the work turns out to be superficial both in terms of phenotypic characterization of NK cells (analyzed only as CD56+) and their functional characterization. Moreover, the low number of donors (N=8 compared to N=41 pts) and the fact that the groups of patients to be compared are unbalanced and under-represented (see T ALL (N=6) and ALL> 50000/mm3 (N=4)) do not allow authors to reach statistical conclusions.

Major points

- Enlarge the number of healthy donors and patients.

- For the cytotoxic and degranulation assays, at least three serial dilutions have to be performed to evaluate NK activity. We suggest aliquots of 100 μl from each NK cell serial dilution containing 2 x 105, 1 x 105 and 0.5 x 105 cells, in triplicates, and aliquots of 100 μl of target cells (1 x 105cells/well) to be added to generate 2:1, 1:1 and 0.5:1 effector—to-target ratio (E:T ratio). In fact, cytotoxicity is more effective at higher E/T ratios, while degranulation responses are stronger at low E/T ratios.

- Together with the frequency (percentage) of NK cells, please report their absolute number.

- In Figure 1B, indicate the p value comparing ALL 10+ and ALL 1-9 that could be the most interesting results you obtained. Eliminate HD or increase their number. Eliminate the comparison between ALL <50000 and >50000 for the same reason.

- Why in figure 2C there is less ALL 1-9 than in figure 1? It could be interesting to understand if they are less numerous compare to ALL 10+ (Fig1B) but more functional.

- Since the paper seeks to clarify why in the Mexican population there is a higher frequency of high-risk relapsing patients and find new risk factors, according to this reviewer opinion, a gene expression analysis, or at least the analysis of NKG2D ligands, could improve the findings of this work and let to more interesting conclusions.

Minor points

- Elimination of leukemia cells relies mainly on their recognition by both T cells and NK cells. Please revise the statement “NK cells are unique lymphocytes that seem to dominate the immune response against leukemia”, otherwise justify it with proper citations from literature.

- Please specify the type of samples collected and the number of cases analyzed (healthy age-matched controls and patients) in the Abstract and in the “Material and Methods” paragraph.

- To estimate the degranulation of NK cells using anti-CD107a mAb, GolgiStop has to be added. Did you? Please clarify the protocol used.

- SAP evaluation in NK cells is innovative. However, according to this reviewer opinion, to see differences among the groups analyzed, the analysis has to be revised indicating the frequency of SAP expressing NK cells and not the expression of SAP inside the NK cell gate.

- In the figure1 legend, correct CD65 with CD56.

- Are they only B-ALL patients in figure3 and 4 as described in the text?

- Why adverse cytogenetic abnormalities have not been considered as risk factors?

- Please better describe the FACS gating strategy. Pay attention to define NK cells as CD56+ cells among CD3- lymphocytes. Specify if and how you discriminate between progenitors and mature lymphocytes. Please show the gating strategy and the representative FACS dot plots/histograms in each figure.

- Figure 1 A. Since T ALL are under represented compared to B ALL, I suggest not to separate B and T ALL.

- In figure 2, correct the legend of the Y-axes: CD107a+ in CD56+CD3- cells. Calculate and report also the number of CD107a+ NK.

- In figure 3, correct the legend of the Y-axes: SAP+ in CD56+CD3- (NK) cells. Calculate also the number of SAP+ NK that could be more informative.

- Figure 5. The statistic is missing.

Reviewer #2: In this study, Valenzuela-Vazquez and collegues have tested NK cell function (degranulation) and expression of SAP in a cohort of 41 pediatric acute leukemia patients in Mexico city. The study cohort is potentially interesting, as it is known that hispanic children have a higher incidence of ALL than non-hispanic children. The study is quite limited in that only degranulation and SAP expression is monitored, but it provides a nice addition to reports on the contribution of NK cells in pediatric ALL patients, for where there are still very few reports.

My main comments are:

- In the introduction you can also comment on the study by de Smith et al in Blood (2014) where they found a correlation between a KIR A haplotype and leukemia risk in hispanic children, this is very relevant information for the current study.

- Materials and methods: Please state the volume of blood drawn, and also include in this section information on the number of patients and healthy controls (even though this is stated in the Results section, it should also be included here).

-Please provide the suplier of the anitbodies used in this study.

- Please explain what is meant by "fold MFI"; is it MFI of SAP in stained samples vs isotype controls? Also MFI is several places misspelled as MIF.

- For degranulation, please clarify how deltaCD107a is calculated; is it the difference between CD107a measured against targets vs NK cells without targets?

- Also clarify in the materials and methods the gating strategy used. For instance on line 255, it is written that a gate was set on CD3+ lymphocytes, did the atuhors mean to say "CD3- lymphocytes"? I assume that the authors have gated cells as CD3-CD56+ throughout the manuscript?

- In the results section, the text describing panel 2C and 2D has been mixed up.

- The figure describing impact of age on NK cell degranulation is missing several data points, please explain why or add the missing data points.

- The authors report that high WBC translate into poor degranulation; can the authors discuss whether this also can be the by lower numbers of NK cells in the PBMC sample that can result in sub-optimal assay?

- I suggest to merge figure 3 and 4 into one figure, and take out the graphs showing boys/girls as there is no difference between different sexes. Also write on the y-axis "%SAP+ NK cells", instead of "% NK SAP+".

- In Figure 3 and 4 there are 18 data points, not 35 data points as stated in the figure legends, please modify.

- Figure legends can be shortened, do not need to repeat methods, they are allready in the materials and methods section.

- The text for Figure 4 does not match the actual figure, please modify.

- Figure 5: Please provide r, and the p-value.

- There are many grammatical and tyopgraphical errors in the manuscript, and I suggest that the authors cirtically re-read the text and correct these. For instance, write "compared to" instead of "regarding".

**********

6. PLOS authors have the option to publish the peer review history of their article (what does this mean?). If published, this will include your full peer review and any attached files.

If you choose “no”, your identity will remain anonymous but your review may still be made public.

**Do you want your identity to be public for this peer review?** For information about this choice, including consent withdrawal, please see our Privacy Policy.

Reviewer #1: No

Reviewer #2: No

[NOTE: If reviewer comments were submitted as an attachment file, they will be attached to this email and accessible via the submission site. Please log into your account, locate the manuscript record, and check for the action link "View Attachments". If this link does not appear, there are no attachment files to be viewed.]

While revising your submission, please upload your figure files to the Preflight Analysis and Conversion Engine (PACE) digital diagnostic tool, https://pacev2.apexcovantage.com/. PACE helps ensure that figures meet PLOS requirements. To use PACE, you must first register as a user. Registration is free. Then, login and navigate to the UPLOAD tab, where you will find detailed instructions on how to use the tool. If you encounter any issues or have any questions when using PACE, please email us at figures@plos.org. Please note that Supporting Information files do not need this step.

# Author comment: Author response to Decision Letter 0

**DOI:** 10.1371/journal.pone.0227314.r002

20 Nov 2019

Professor Joerg Heber

Editor-in-Chief

Plos One

Dear Dr. Heber:

You will find enclosed the revised version of manuscript PONE-D-19-20648, which we would like considered for publication in PlosOne.

We are pleased that the Editors and Reviewers were very supportive of our manuscript. As requested, we have addressed the queries of the Reviewers, by adding additional data and clarifications in the text. The corresponding changes in the text are highlighted in yellow. As a result of these various changes, we believe that the manuscript is much improved. We have also modified the manuscript according to the PlosOne format instructions and publication policies. 

Below is a point-by-point response to the Reviewers’ comments.

Major Points

Reviewer #1:

1. Enlarge the number of healthy donors and patients.

We agree with reviewer that the number of controls and patients is unbalanced. In order to improve the number of healthy controls analyzed, we have included 6 more age-matched healthy controls to the study. However, it is important to mention that statistical conclusions are possible taking into account that with the sample size analyzed of cases and controls, the differences observed were statistically significant with an adequate statistical power. Considering the sample size here analyzed, if the pooled variance would have been so small as 5 or so high as 20; therefore, the sample size used has a power to detect differences of 5% among the groups with a power of 94.38%. If the difference between the two groups would have been 10% or higher, the power to detect differences is of 100%. In the case of analyses of subgroups with 8 patients, differences of 5% and a pooled variance of 5% too, the statistical power was of 98.8%. For differences of 7% among subgroups, the power was of 100%. When we compared subgroups of 5 patients with differences of 5% among groups and the pooled variance was 5% too, the power was of 89.75%. When a differences of 10% was observed, the statistical power was of 100%.

On the other hand, it is important to mention that a lower number of controls is not an impediment for made comparisons between cases and controls. Although we agree that the inclusion of more controls could enhance the statistical power, the associations remained statistically significant after the inclusion of more controls. 

2. For the cytotoxic and degranulation assays, at least three serial dilutions have to be performed to evaluate NK activity. We suggest aliquots of 100 μl from each NK cell serial dilution containing 2 x 105, 1 x 105 and 0.5 x 105 cells, in triplicates, and aliquots of 100 μl of target cells (1 x 105cells/well) to be added to generate 2:1, 1:1 and 0.5:1 effector—to-target ratio (E:T ratio). In fact, cytotoxicity is more effective at higher E/T ratios, while degranulation responses are stronger at low E/T ratios.

We concur with the Reviewer that cytotoxic assays are also informative in evaluating NK cell function, however the paucity of peripheral blood available from each patient it makes difficult to performed both assays (specific lysis and degranulation assays) in all samples. Moreover, degranulation assays is a well-known standard method that allow to assess NK cell-mediated cytotoxicity and this method is broadly used not only in basic approaches to assess NK cell function but also for diagnosis of pathologies related to an abnormal NK cell function. We have included the proper references in Materials and methods.

3. Together with the frequency (percentage) of NK cells, please report their absolute number.

We agree with reviewer that this is an important information, unfortunately this information was not available for all samples. 

4. In Figure 1B, indicate the p value comparing ALL 10+ and ALL 1-9 that could be the most interesting results you obtained. Eliminate HD or increase their number. Eliminate the comparison between ALL <50000 and >50000 for the same reason.

The requested p value has been indicated in revised Figure 1. In addition, we have increased the number of HC as suggested. The comparison between ALL <50000 and >50000 has been eliminated in the new figure 1 as requested.

5. Why in figure 2C there is less ALL 1-9 than in figure 1? It could be interesting to understand if they are less numerous compare to ALL 10+ (Fig1B) but more functional.

This is an excellent point. This information has been corrected accordingly. These data are mentioned on p. 13, lines 298-300 and p. 15, lines 325-327

6. Since the paper seeks to clarify why in the Mexican population there is a higher frequency of high-risk relapsing patients and find new risk factors, according to this reviewer opinion, a gene expression analysis, or at least the analysis of NKG2D ligands, could improve the findings of this work and let to more interesting conclusions.

We agree that a gene expression analysis, or at least the analysis of NKG2D ligands, could improve the findings of this work, however we considered that the gene expression analysis was not the scope of this manuscript or it will be part of other manuscript.

Minor Points

7. Elimination of leukemia cells relies mainly on their recognition by both T cells and NK cells. Please revise the statement “NK cells are unique lymphocytes that seem to dominate the immune response against leukemia”, otherwise justify it with proper citations from literature.

We agree with the reviewer; the statement has been modified accordingly throughout the text. 

8. Please specify the type of samples collected and the number of cases analyzed (healthy age-matched controls and patients) in the Abstract and in the “Material and Methods” paragraph.

We have included this information in the revised manuscript as requested by the reviewer. This information is mentioned on p. 4, lines 83-84, and p.4, lines 87-88. 

9. To estimate the degranulation of NK cells using anti-CD107a mAb, GolgiStop has to be added. Did you? Please clarify the protocol used.

We agree with the reviewer that the addition of GolgiStop is included in some protocols, however there are other that not include the use of GolgiStop, as is in this case. This information has been clarified in Materials and Methods. In addition, the proper references have been included. 

10. SAP evaluation in NK cells is innovative. However, according to this reviewer opinion, to see differences among the groups analyzed, the analysis has to be revised indicating the frequency of SAP expressing NK cells and not the expression of SAP inside the NK cell gate.

This point was included in the analysis as suggested by reviewer.

11. In the figure 1 legend, correct CD65 with CD56.

This was corrected in the text as indicated. 

12. Are they only B-ALL patients in figure 3 and 4 as described in the text?

For figures 3 and 4 only B-ALL patients were included as indicated in the text.

13. - Why adverse cytogenetic abnormalities have not been considered as risk factors?

This is an excellent point; however, gene rearrangements are found in only 17.7% of the patients with acute lymphoblastic leukemia from Mexico City. This information has been included in the discussion to clarified the point indicated by the reviewer. p. 21, lines 466-469.

14. Please better describe the FACS gating strategy. Pay attention to define NK cells as CD56+ cells among CD3- lymphocytes. Specify if and how you discriminate between progenitors and mature lymphocytes. 

We have better described these points in the revised manuscript as indicated by the reviewer. Since all analysis were performed in mononuclear cells form peripheral blood, we did not discriminate between progenitors and mature lymphocytes.

15. Please show the gating strategy and the representative FACS dot plots/histograms in each figure.

A representative FACS plots/histogram has been included in each figure as suggested by the reviewer. 

16. Figure 1 A. Since T ALL are underrepresented compared to B ALL, I suggest not to separate B and T ALL.

This is an important suggestion, however we think that data is important as this information has not been published previously.

17. In figure 2, correct the legend of the Y-axes: CD107a+ in CD56+CD3- cells. Calculate and report also the number of CD107a+ NK.

The legend has been corrected as suggested by the reviewer. Unfortunately, we did not analyze the absolute number of NK cells in this study or this information was not available for all samples. 

18. In figure 3, correct the legend of the Y-axes: SAP+ in CD56+CD3- (NK) cells. Calculate also the number of SAP+ NK that could be more informative.

The legend has been corrected as suggested by the reviewer. Unfortunately, we did not analyze the absolute number of NK cells in this study, or this information was not available for all samples. 

19. Figure 5. The statistic is missing.

The statistic has been incorporated in corresponding figure as indicated by reviewer. 

Reviewer #2:

1. In the introduction you can also comment on the study by de Smith et al in Blood (2014) where they found a correlation between a KIR A haplotype and leukemia risk in hispanic children, this is very relevant information for the current study.

This is an excellent point. The study referred by the reviewer has been commented in the introduction as indicated in p. 8, lines 173-175. 

2. Materials and methods: Please state the volume of blood drawn, and also include in this section information on the number of patients and healthy controls (even though this is stated in the Results section, it should also be included here).

We have included this information as requested by reviewer. 

3. Please provide the supplier of the antibodies used in this study.

This information has been included in Materials and Methods.

4. Please explain what is meant by "fold MFI"; is it MFI of SAP in stained samples vs isotype controls? Also MFI is several places misspelled as MIF.

This information has been corrected. 

5. For degranulation, please clarify how deltaCD107a is calculated; is it the difference between CD107a measured against targets vs NK cells without targets?

This information has been clarified in Materials and Methods. See p. 12, lines 265-268

6. Also clarify in the materials and methods the gating strategy used. For instance on line 255, it is written that a gate was set on CD3+ lymphocytes, did the authors mean to say "CD3- lymphocytes"? I assume that the authors have gated cells as CD3-CD56+ throughout the manuscript?

We appreciate this comment from reviewer. This point has been clarified in the Materials and Methods section. 

7. In the results section, the text describing panel 2C and 2D has been mixed up.

This point has been corrected accordingly.

8. The figure describing impact of age on NK cell degranulation is missing several data points, please explain why or add the missing data points.

This point has been corrected.

9. The authors report that high WBC translate into poor degranulation; can the authors discuss whether this also can be the by lower numbers of NK cells in the PBMC sample that can result in sub-optimal assay?

This is an excellent point by the reviewer. Whereas this possibility can not be formally excluded, we did not find a significant difference in the percentages of NK cells between patients with high or low WBC. 

10. I suggest to merge figure 3 and 4 into one figure, and take out the graphs showing boys/girls as there is no difference between different sexes. Also write on the y-axis "%SAP+ NK cells", instead of "% NK SAP+".

We appreciate the suggestions by the reviewer. The y-axis has been corrected, and graphs showing boys/girls has been eliminated. Merge of figures 3 and 4 was not possible since new data was incorporated as suggested by reviewer #1.

11. In Figure 3 and 4 there are 18 data points, not 35 data points as stated in the figure legends, please modify.

The legend has been modified accordingly.

12. Figure legends can be shortened, do not need to repeat methods, they are already in the materials and methods section.

Figures legends were shortened as suggested by reviewer.

13. The text for Figure 4 does not match the actual figure, please modify.

The Figure 4 and text has been corrected. 

14. Figure 5: Please provide r, and the p-value.

These values have been incorporated in figure 5.

15. There are many grammatical and typographical errors in the manuscript, and I suggest that the authors critically re-read the text and correct these. For instance, write "compared to" instead of "regarding".

We appreciate the comments by the reviewer. Grammatical and typographical errors have been revised and corrected.

Attachment Submitted filename: Response to Reviewers.docx Click here for additional data file.

# Peer review: Decision Letter 1

**DOI:** 10.1371/journal.pone.0227314.r003

**Authors:** Francesco Bertolini

3 Dec 2019

PONE-D-19-20648R1

Functional characterization of NK cells in Mexican pediatric patients with acute lymphoblastic leukemia: Report from the Mexican Interinstitutional Group for the Identification of the Causes of Childhood Leukemia.

PLOS ONE

Dear Dr Cruz-Munoz,

Thank you for submitting your manuscript to PLOS ONE. After careful consideration, we feel that it has merit but does not fully meet PLOS ONE’s publication criteria as it currently stands. Therefore, we invite you to submit a revised version of the manuscript that addresses the points raised during the review process by Reviewer #1.

We would appreciate receiving your revised manuscript by Jan 17 2020 11:59PM. When you are ready to submit your revision, log on to https://www.editorialmanager.com/pone/ and select the 'Submissions Needing Revision' folder to locate your manuscript file.

If you would like to make changes to your financial disclosure, please include your updated statement in your cover letter.

To enhance the reproducibility of your results, we recommend that if applicable you deposit your laboratory protocols in protocols.io, where a protocol can be assigned its own identifier (DOI) such that it can be cited independently in the future. For instructions see: http://journals.plos.org/plosone/s/submission-guidelines#loc-laboratory-protocols

Please include the following items when submitting your revised manuscript:

- A rebuttal letter that responds to each point raised by the academic editor and reviewer(s). This letter should be uploaded as separate file and labeled 'Response to Reviewers'.
- A marked-up copy of your manuscript that highlights changes made to the original version. This file should be uploaded as separate file and labeled 'Revised Manuscript with Track Changes'.
- An unmarked version of your revised paper without tracked changes. This file should be uploaded as separate file and labeled 'Manuscript'.

Please note while forming your response, if your article is accepted, you may have the opportunity to make the peer review history publicly available. The record will include editor decision letters (with reviews) and your responses to reviewer comments. If eligible, we will contact you to opt in or out.

We look forward to receiving your revised manuscript.

Kind regards,

Francesco Bertolini, MD, PhD

Academic Editor

PLOS ONE

[Note: HTML markup is below. Please do not edit.]

Reviewers' comments:

Reviewer's Responses to Questions

**Comments to the Author**

1. If the authors have adequately addressed your comments raised in a previous round of review and you feel that this manuscript is now acceptable for publication, you may indicate that here to bypass the “Comments to the Author” section, enter your conflict of interest statement in the “Confidential to Editor” section, and submit your "Accept" recommendation.

Reviewer #1: All comments have been addressed

Reviewer #2: All comments have been addressed

**********

2. Is the manuscript technically sound, and do the data support the conclusions?

The manuscript must describe a technically sound piece of scientific research with data that supports the conclusions. Experiments must have been conducted rigorously, with appropriate controls, replication, and sample sizes. The conclusions must be drawn appropriately based on the data presented. 

Reviewer #1: Yes

Reviewer #2: Yes

**********

3. Has the statistical analysis been performed appropriately and rigorously? 

Reviewer #1: Yes

Reviewer #2: Yes

**********

4. Have the authors made all data underlying the findings in their manuscript fully available?

The PLOS Data policy requires authors to make all data underlying the findings described in their manuscript fully available without restriction, with rare exception (please refer to the Data Availability Statement in the manuscript PDF file). The data should be provided as part of the manuscript or its supporting information, or deposited to a public repository. For example, in addition to summary statistics, the data points behind means, medians and variance measures should be available. If there are restrictions on publicly sharing data—e.g. participant privacy or use of data from a third party—those must be specified.

Reviewer #1: Yes

Reviewer #2: Yes

**********

5. Is the manuscript presented in an intelligible fashion and written in standard English?

PLOS ONE does not copyedit accepted manuscripts, so the language in submitted articles must be clear, correct, and unambiguous. Any typographical or grammatical errors should be corrected at revision, so please note any specific errors here.

Reviewer #1: Yes

Reviewer #2: Yes

**********

6. Review Comments to the Author

Please use the space provided to explain your answers to the questions above. You may also include additional comments for the author, including concerns about dual publication, research ethics, or publication ethics. (Please upload your review as an attachment if it exceeds 20,000 characters)

Reviewer #1: I thanks Authors for accurately correct the manuscript answering all the questions.

Some suggestions:

Minor

line 302 please use "lower" instead of "abnormal".

line 305 percentages of nk cell in the lymphocyte gates.

Fig 2 c, d, e. You can appreciate two clusters. Please be sure that data are correct and not due to acquisition byass. Same day of acquisition?

Major

line 368 "significant difference" it is right but p value is 0.0468. Please pay attention to not overestimate data and conclusions.

line 450 "severe impaired nk cytotoxicity" is not a correct definition. p value is 0.02 and sample size is little and this could bring to uncertain conclusions. The same at line 499, please eliminate "severely".

Reviewer #2: (No Response)

**********

7. PLOS authors have the option to publish the peer review history of their article (what does this mean?). If published, this will include your full peer review and any attached files.

If you choose “no”, your identity will remain anonymous but your review may still be made public.

**Do you want your identity to be public for this peer review?** For information about this choice, including consent withdrawal, please see our Privacy Policy.

Reviewer #1: No

Reviewer #2: No

[NOTE: If reviewer comments were submitted as an attachment file, they will be attached to this email and accessible via the submission site. Please log into your account, locate the manuscript record, and check for the action link "View Attachments". If this link does not appear, there are no attachment files to be viewed.]

While revising your submission, please upload your figure files to the Preflight Analysis and Conversion Engine (PACE) digital diagnostic tool, https://pacev2.apexcovantage.com/. PACE helps ensure that figures meet PLOS requirements. To use PACE, you must first register as a user. Registration is free. Then, login and navigate to the UPLOAD tab, where you will find detailed instructions on how to use the tool. If you encounter any issues or have any questions when using PACE, please email us at figures@plos.org. Please note that Supporting Information files do not need this step.

# Author comment: Author response to Decision Letter 1

**DOI:** 10.1371/journal.pone.0227314.r004

10 Dec 2019

December 5, 2019

Professor Joerg Heber

Editor-in-Chief

Plos One

Dear Dr. Heber:

You will find enclosed the revised version of manuscript PONE-D-19-20648, which we would like considered for publication in Plos One.

We are very pleased that the Editors and Reviewers were very supportive of our manuscript. As requested, we have addressed the queries of the Reviewer #1, by doing clarifications in the text. The corresponding changes in the text are highlighted in yellow. As a result of these changes, we believe that the manuscript is improved. We have also modified the manuscript according to the PlosOne format instructions and publication policies. 

Below is a point-by-point response to the comments of the Reviewer #1.

Reviewer #1:

Minor suggestions

1. line 302 please use "lower" instead of "abnormal"

We have use lower instead of abnormal in the corresponding line.

2. line 305 percentages of nk cell in the lymphocyte gates.

We have included in the text the suggestion made by the reviewer.

3. Fig 2 c, d, e. You can appreciate two clusters. Please be sure that data are correct and not due to acquisition byass. Same day of acquisition?

This is an interesting point. We agree with the reviewer that two cluster can be observed in some graphics, however we have checked all data and we are certainly that information is correct and no due to acquisition artefacts. We did not acquire all samples the same day. To ensure the reliability of our results for immunophenotyping and functional assays, all experiments were analyzed in a flow cytometer FACS ARIA III (BD) with a biannual maintenance, inspection and correction service. Additionally, the quality of measures is sustained by daily quality controls monitoring overall flow cytometric performance (laser alignment and fluorescence stability). 

Major suggestions

4. line 368 "significant difference" it is right but p value is 0.0468. Please pay attention to not overestimate data and conclusions.

We agree with the reviewer. We have put attention in order to not overestimate data and conclusions by modifying the respective phrase in the text. 

“Although we did observe a significant difference only in patients with standard-risk compared to healthy controls, we did not observe any difference when healthy donors were compared to those ALL patients in high-risk group…”

5. line 450 "severe impaired nk cytotoxicity" is not a correct definition. p value is 0.02 and sample size is little and this could bring to uncertain conclusions. The same at line 499, please eliminate "severely".

We agree with the reviewer. We have made the suggestions made by the reviewer by not using the word “severe” or “severely” throughout the text.

“Moreover, patients presenting a leukocyte count ≥ 50,000xmm3 displayed a severe reduction in NK-cell mediated cytotoxicity….” 

“In addition, NK cell function was observed to be severely compromised in patients with a leukocyte count….”

“Interestingly, NK cell cytotoxicity was severely compromised in those B-ALL patients with WBC over….”

“Finally, a severe impaired NK cell-mediated cytotoxicity was also observed in those B-ALL patients with WBC over 50,000xmm3 compared to B-ALL patients with WBC below 50,000xmm3….”

“The finding that those patients with WBC over 50,000xmm3 displayed a severely reduced NK cell lysis towards K562….”

“Moreover, NK cell-mediated cytotoxicity was severely compromised in those patients with a leukocyte count ≥50,000xmm3 where also it was found a decreased expression of SAP regarding patients with a leukocyte count < 50,000xmm3…”

We thank you and the Reviewers for your support and constructive comments. We hope that our revised in now suitable for publication in Plos One.

Sincerely,

Mario E. Cruz-Muñoz

Juan Manuel Mejía-Aranguré

Attachment Submitted filename: Response to Reviewers.docx Click here for additional data file.

# Peer review: Decision Letter 2

**DOI:** 10.1371/journal.pone.0227314.r005

**Authors:** Francesco Bertolini

17 Dec 2019

Functional characterization of NK cells in Mexican pediatric patients with acute lymphoblastic leukemia: Report from the Mexican Interinstitutional Group for the Identification of the Causes of Childhood Leukemia.

PONE-D-19-20648R2

Dear Dr. Cruz-Munoz,

We are pleased to inform you that your manuscript has been judged scientifically suitable for publication and will be formally accepted for publication once it complies with all outstanding technical requirements.

Within one week, you will receive an e-mail containing information on the amendments required prior to publication. When all required modifications have been addressed, you will receive a formal acceptance letter and your manuscript will proceed to our production department and be scheduled for publication.

Shortly after the formal acceptance letter is sent, an invoice for payment will follow. To ensure an efficient production and billing process, please log into Editorial Manager at https://www.editorialmanager.com/pone/, click the "Update My Information" link at the top of the page, and update your user information. If you have any billing related questions, please contact our Author Billing department directly at authorbilling@plos.org.

If your institution or institutions have a press office, please notify them about your upcoming paper to enable them to help maximize its impact. If they will be preparing press materials for this manuscript, you must inform our press team as soon as possible and no later than 48 hours after receiving the formal acceptance. Your manuscript will remain under strict press embargo until 2 pm Eastern Time on the date of publication. For more information, please contact onepress@plos.org.

With kind regards,

Francesco Bertolini, MD, PhD

Academic Editor

PLOS ONE

Additional Editor Comments (optional):

Reviewers' comments:

Reviewer's Responses to Questions

**Comments to the Author**

1. If the authors have adequately addressed your comments raised in a previous round of review and you feel that this manuscript is now acceptable for publication, you may indicate that here to bypass the “Comments to the Author” section, enter your conflict of interest statement in the “Confidential to Editor” section, and submit your "Accept" recommendation.

Reviewer #1: All comments have been addressed

Reviewer #2: (No Response)

**********

2. Is the manuscript technically sound, and do the data support the conclusions?

The manuscript must describe a technically sound piece of scientific research with data that supports the conclusions. Experiments must have been conducted rigorously, with appropriate controls, replication, and sample sizes. The conclusions must be drawn appropriately based on the data presented. 

Reviewer #1: (No Response)

Reviewer #2: Yes

**********

3. Has the statistical analysis been performed appropriately and rigorously? 

Reviewer #1: (No Response)

Reviewer #2: Yes

**********

4. Have the authors made all data underlying the findings in their manuscript fully available?

The PLOS Data policy requires authors to make all data underlying the findings described in their manuscript fully available without restriction, with rare exception (please refer to the Data Availability Statement in the manuscript PDF file). The data should be provided as part of the manuscript or its supporting information, or deposited to a public repository. For example, in addition to summary statistics, the data points behind means, medians and variance measures should be available. If there are restrictions on publicly sharing data—e.g. participant privacy or use of data from a third party—those must be specified.

Reviewer #1: (No Response)

Reviewer #2: Yes

**********

5. Is the manuscript presented in an intelligible fashion and written in standard English?

PLOS ONE does not copyedit accepted manuscripts, so the language in submitted articles must be clear, correct, and unambiguous. Any typographical or grammatical errors should be corrected at revision, so please note any specific errors here.

Reviewer #1: (No Response)

Reviewer #2: Yes

**********

6. Review Comments to the Author

Please use the space provided to explain your answers to the questions above. You may also include additional comments for the author, including concerns about dual publication, research ethics, or publication ethics. (Please upload your review as an attachment if it exceeds 20,000 characters)

Reviewer #1: (No Response)

Reviewer #2: I have no further comments, as my concerns were answered after the first revision. THe study is a Nice contribution to the role of NK cells in pediatric acute leukemia patients.

**********

7. PLOS authors have the option to publish the peer review history of their article (what does this mean?). If published, this will include your full peer review and any attached files.

If you choose “no”, your identity will remain anonymous but your review may still be made public.

**Do you want your identity to be public for this peer review?** For information about this choice, including consent withdrawal, please see our Privacy Policy.

Reviewer #1: No

Reviewer #2: No

# Editor report: Acceptance letter

**DOI:** 10.1371/journal.pone.0227314.r006

**Authors:** Francesco Bertolini

23 Dec 2019

PONE-D-19-20648R2 

Functional characterization of NK cells in Mexican pediatric patients with acute lymphoblastic leukemia: Report from the Mexican Interinstitutional Group for the Identification of the Causes of Childhood Leukemia. 

Dear Dr. Cruz-Munoz:

I am pleased to inform you that your manuscript has been deemed suitable for publication in PLOS ONE. Congratulations! Your manuscript is now with our production department. 

If your institution or institutions have a press office, please notify them about your upcoming paper at this point, to enable them to help maximize its impact. If they will be preparing press materials for this manuscript, please inform our press team within the next 48 hours. Your manuscript will remain under strict press embargo until 2 pm Eastern Time on the date of publication. For more information please contact onepress@plos.org.

For any other questions or concerns, please email plosone@plos.org. 

Thank you for submitting your work to PLOS ONE.

With kind regards,

PLOS ONE Editorial Office Staff

on behalf of

Dr. Francesco Bertolini 

Academic Editor

PLOS ONE
